# Prevalence of Catalase (-21 A/T) Gene Variant in South Indian (Tamil) Population

**DOI:** 10.1155/2014/894237

**Published:** 2014-06-26

**Authors:** A. Lourdhu Mary, K. Nithya, W. Isabel, T. Angeline

**Affiliations:** PG & Research Department of Zoology and Biotechnology, Lady Doak College, Madurai, Tamil Nadu 625 002, India

## Abstract

Catalase, an endogenous antioxidant enzyme, is responsible for regulating reactive species levels. Several epidemiologic studies have suggested that single nucleotide polymorphism in catalase gene may be associated with many diseases. The genotype of CAT (-21 A/T) point mutation in promoter region of catalase gene was determined by polymerase chain based restriction fragment length polymorphism analysis in the DNA of 100 healthy volunteers. The frequency of CAT (-21 A/T) gene polymorphism AA, AT, and TT genotypes was found to be 7, 23, and 70 percent, respectively. The mutant “T” allele frequency was found to be 0.82 among the south Indian (Tamil) population. Chi square analysis showed that the study population lies within the Hardy-Weinberg equilibrium. The wild type genotype (AA) was found to be very low (7%) and the mutant genotype (AT/TT) was found to be more prevalent (93%) among the south Indian population. This suggests that the high prevalence of mutant genotype may increase the susceptibility to oxidative stress associated diseases.

## 1. Introduction 

Excessive oxidative stress has been implicated in the pathogenesis of several diseases. In humans, the antioxidant enzymes pathway is available to protect against the toxicity caused by oxidative stress. Many of the antioxidant genes are known to be polymorphic, which may alter the enzyme activity [1].

Catalase is an antioxidant enzyme that plays a major role in controlling hydrogen peroxide concentration in human cells. It decomposes H_2_O_2_ into H_2_O and O_2_, thereby protecting the cells from oxidative stress. It has been suggested that functional polymorphism in the gene encoding catalase enzyme affects the enzyme activity, thereby reducing the protection against oxidative stress [2]. Apart from the decomposition of H_2_O_2_, catalase inactivates many environmental mutagens. It also prevents chromosomal aberration caused by hypoxanthine/xanthine oxidase in Chinese hamster cells [3]. Several studies have reported on the alterations in catalase activity in hypertension [4], cancer [5], diabetes, nephropathy [6, 7], and other diseases accompanied by oxidative stress.

Catalase is a homotetramer of 220–230 kDa mass having four heme groups in its structure [8, 9]. The CAT gene is localized on chromosome 11p 13.31 consisting of 13 exons and 12 introns. CAT (-21 A/T) variant represents the A to T substitution in the promoter region, which is located inside the promoter region just proximal to the start site [8]. The mutant allele frequency may vary based on the differences in the study population's race and ethnicity and also on the underlying characters.

This study, which has been focused on the genetic variant -21 A/T of the catalase enzyme, was the first kind regarding the prevalence of catalase gene variant in healthy individuals among south Indian Tamil population. The objective was to find out the prevalence of mutant allele frequency of the evaluated catalase (-21 A/T) gene polymorphism in healthy individuals among the south Indian population.

## 2. Materials and Methods

### 2.1. Study Design

A total of 100 healthy controls with no history of cardiovascular disease, diabetes, hypertension, cancer, or any infectious diseases and belonged to south Indian Tamil ethnicity were included for the study. The samples from the volunteers were collected into EDTA coated tubes and the informed consent was obtained. The present study was approved by the ethical committee and biosafety committee of the institution.

### 2.2. DNA Extraction

Genomic DNA was extracted from the frozen blood by phenol-chloroform method [10]. For DNA extraction, 500 *μ*L of blood was used and the isolated DNA dissolved in TE was stored at −20°C. The quality of the DNA was checked in 0.7 percent agarose (Hi-Media, Mumbai) gel electrophoresis and quantified using UV spectrophotometry (Hitachi, Japan).

### 2.3. PCR Analysis

**Figure 1:**
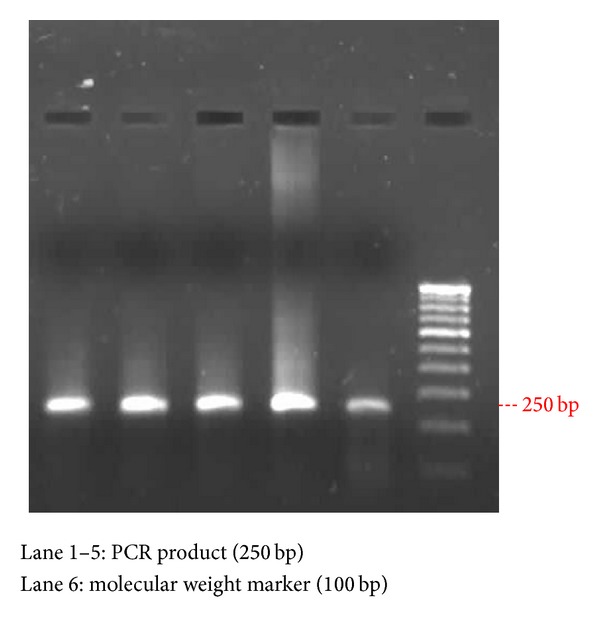
PCR analysis of the catalase gene.

PCR analysis was carried out using a thermal cycler (Eppendorf Master Cycler, Germany). Approximately 120 ng of genomic DNA was incubated in a total reaction mixture of 50 *μ*L containing both the forward primer 5′-AATCAGAAGGCAGTCCTCCC-3′ and the reverse primer 5′-TCGGGGAGCACAGAGTGTAC-3′ (~70 picomoles) (GenScript Corp., USA), 200 *μ*M deoxynucleotide triphosphate, 10X PCR buffer of* p*H-8.3 containing 15 mM MgCl_2_, and 5 units of* Taq *DNA polymerase (New England Biolabs, Beverly) [11]. DNA was initially denatured at 95°C for 4 min prior to amplification. A 250 bp of catalase gene was amplified using the following conditions with 30 cycles consisting of 1 min denaturation at 94°C, 40 sec annealing at 61°C, and 1 min extension at 72°C. The final extension included 5 mins at 72°C and the PCR products were confirmed by ethidium bromide stained 1.2 percent agarose gel and viewed in the UV-transilluminator (Figure 1).

### 2.4. Restriction Enzyme Analysis

**Figure 2:**
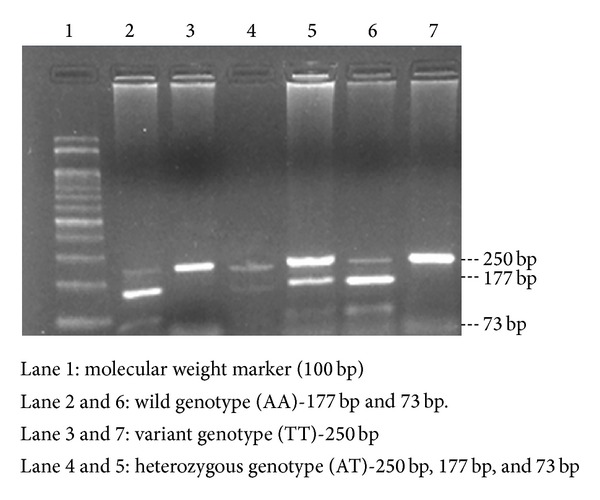
Restriction band pattern of catalase (-21 A/T) gene variant.

Restriction digestion was performed in a total volume of 10 *μ*L consisting of 5 *μ*L amplicon, 1 *μ*L NE buffer (50 mM potassium acetate, 20 mM Tris-acetate, 10 mM magnesium acetate, and 1 mM dithiothreitol* p*H-7.9 at 25°C), and 10 U of* Hinf*1 enzyme (Fermentas Life Sciences, Germany) [11]. Samples were incubated for 6-7 hrs at 37°C and the digested PCR products were resolved in 2 percent agarose gel electrophoresis stained with ethidium bromide and separated bands were observed using gel documentation system (Figure 2). The A → T mutation at nucleotide 250 bp abolishes a* Hinf*1 restriction site (T allele), which otherwise forms 177 and 73 bp fragments (A allele) when treated with* Hinf*1. The catalase gene (-21) polymorphism abolishes a* Hinf*1 site and so there will not be any cleavage of the PCR products (250 bp) and if there is no mutation (normal genotype-AA), the band pattern in the gel will show the cleavage of the fragment into 177 bp and 73 bp. In the case of heterozygous genotype (AT), all the three types of fragments (250 bp, 177 bp, and 73 bp) will be observed in the gel.

### 2.5. Sequencing of the CAT Gene

The PCR product obtained was subjected to DNA sequencing which was carried out by Sanger's sequencing method (Synergy scientific services, Chennai) [12]. The DNA sequencing was done to check and confirm whether the amplified product was CAT gene sequence or not. The obtained sequence was then subjected to the BLAST N [13] analysis to study the homology sequence of the amplified product.

## 3. Results and Discussion

The -21 A/T (rs7943316) polymorphism in CAT gene gains its importance mainly by its position being close to transcription start site, or at sites where the transcription factors bind [14]. In the present study, one hundred healthy volunteers were analysed for SNP in catalase -21 A/T gene polymorphism. It has been observed that only 7% of the individuals have AA genotype and 70% of individuals have TT genotype and the remaining 23% of individuals have AT heterozygous mutant genotype (Table 1). The highest TT genotype (70%) of -21 A/T catalase gene polymorphism was observed among south Indians and the lowest TT genotype (8%) was observed among Chinese population [2].

When BLAST N analysis was performed with the DNA sequence of PCR product (250 bp), 98 percent homology was found between the CAT gene and the submitted DNA sequence. The “T” allele frequency observed in the present study was found to be very high (0.82) when compared to that of Finnish population (0.58) [11], Czechs (0.4) [15], Europeans (0.31) [16], and Chinese (0.4, 0.2) [2, 5] (Table 2). A study conducted among Indian population with diabetes mellitus has reported that 80% of the patients were of TT genotype and 20% were of AT genotype. AA genotype was not observed among patients and healthy individuals were not included in their study. The study also found lower activity of scavenger enzymes in patients with diabetes mellitus [9]. However, decreased catalase activity can be influenced by the disease itself and not by gene polymorphism.

Catalase is one of the major enzyme components of cell defense against oxidative stress and it has been hypothesized that the polymorphism of -21 A/T CAT reduces the antioxidant capacity and may serve as a risk factor for oxidative stress associated diseases. The association between SOD1-251 A/G, CAT-21 A/T, and GPX1-198C/T antioxidant gene polymorphisms in the risk of cataract was reported among Chinese population and was found to have no significant association in CAT-21 A/T and GPX1-198C/T polymorphisms between the controls and patients. However, SOD1-251 A/G polymorphism was found to be associated with an increased risk of cataract [2].

Experimental and clinical studies conducted among essential hypertension and cerebral stroke patients suggested that the polymorphism -21 A/T CAT was associated with increased risk and was found to play an important role in pathogenesis [17]. Similar study conducted among Russian population reported that there was an association between polymorphism -21 A/T of the catalase gene and bronchial asthma. The study also reported that cigarette smoking and fruits and vegetables intake have potentially inverse modifying influences on the risk of asthma [18]. In contrast few studies reported that the catalase gene polymorphism was not related to the risk of cardiovascular diseases among type 2 diabetes mellitus in Finnish population [11] and also among type 1 diabetes mellitus patients in Czech population [15].

Even though, catalase was not essential for some cell types under normal conditions, it was reported to play an important role in the adaptive response to oxidative stress [19, 20]. Earlier studies reported that the catalase activity was found to be higher among cancer patients. However it has been reported that an adaptive increase of catalase activity may not be sufficient for inactivation of reactive oxygen species [21]. Decreased catalase gene expression due to the presence of mutant allele may further decrease the activity of catalase enzyme. The presence of insufficient antioxidant enzyme system may increase the susceptibility to oxidative stress-associated diseases among south Indian population.

Apart from -21 A/T polymorphism of the catalase gene, another polymorphism (-262 C/T) in the promoter region has been shown to influence transcription factor binding and correlates well with blood catalase levels [22]. There has been a well-established association between the type 1 diabetic neuropathy patients and the latter polymorphism in the Russian population [23].

In conclusion, the present study demonstrates that CAT (-21 A/T) SNP is more prevalent among the south Indian population. Further study should be conducted to find out whether there is alteration in catalase activity in the individuals with mutant genotype for CAT (-21 A/T) polymorphism among healthy individuals and also need to be correlated with any disease associated with oxidative stress such as cancer, diabetes, and coronary artery diseases. The other polymorphisms within the same gene should also be studied for better understanding of the role of catalase gene in the mediation of oxidative stress response.

## Figures and Tables

**Table 1:** Catalase gene -21 A/T polymorphism-genotype and allele frequency in healthy controls.

Genotype/Allele	Healthy controls (*n* = 100)
Genotypes (-21 A/T)	
AA	7
AT	23
TT	70
Alleles	
A	0.18
T	0.82

**Table 2:** Prevalence of the catalase gene -21 A/T polymorphism in healthy controls.

Country	Ethnic population	Number of subjects	Genotype frequency			T allele frequency
			A/A	A/T	T/T	
India	South Indian (present study)	100	7	23	70	0.82
Brazil [24]	Brazilian	124	18.4	42.4	39.2	0.604
Finland [11]	Finnish	245	18	48	34	0.58
Brazil [25]	Brazilian	135	22	39	39	0.57
Czech Republic [15]	Czechs	180	37	48	15	0.40
China [5]	Chinese	848	32	56	12	0.40
China [2]	Chinese	386	54	38	8	0.272
New Zealand [16]	Europeans	190	48	42	10	0.31
